# Supplementary material for: Bogong moths use a stellar compass for long-distance navigation at night
Source: Nature. 2025 Jun 18;643(8073):994–1000. doi: 10.1038/s41586-025-09135-3 (PMC12286839; doi:10.1038/s41586-025-09135-3)
Supplement: Supplementary file 1 — The underlying population-level statistics for each population of moths tested in each season during 2018 and 2019 under seasonally appropriate naturalistic projected night skies at 0° (natural orientation) or rotated 180°, or under randomized starry night skies (which form the basis of the results shown in Fig. 3). [file 41586_2025_9135_MOESM1_ESM.pdf]

nature portfolio

https://doi.org/10.1038/s41586-025-09135-3

Supplementary information

Bogong moths use a stellar compass for long-distance navigation at night

In the format provided by the authors and unedited

Supplementary Table 1

Statistical analysis of all tested subsamples of moths shown in Main Paper Figure 3

	Spring 2018 A	Spring 2018 B	Spring 2018 C	Autumn 2018 D	Autumn 2018 E	Autumn 2018 F	Spring 2019 G	Spring 2019 H	Spring 2019 I	Autumn 2019 J	Autumn 2019 K	Autumn 2019 L
**Moth N° (n)**	20	24	15	31	28	24	50	44	43	23	28	25
**MV (μ)**	176°	18°	330°	337°	144°	325°	155°	337°	26°	339°	197°	209°
**GMV (α)**	187°	15°	328°	341°	148°	12°	159°	340°	22°	342°	197°	222°
**Length MV (r)**	0.524	0.544	0.156	0.633	0.504	0.159	0.342	0.456	0.084	0.474	0.364	0.224
**CV**	0.476	0.456	0.844	0.367	0.496	0.841	0.658	0.544	0.916	0.526	0.636	0.776
**CSD**	65°	63°	110°	55°	67°	110°	84°	72°	128°	70°	81°	99°
**95% CI (-/+) for μ**	145°<br>208°	351°<br>46°	*****<br>*****	317°<br>357°	116°<br>171°	224°<br>66°	124°<br>187°	312°<br>2°	242°<br>170°	306°<br>11°	157°<br>236°	140°<br>279°
**99% CI (-/+) for μ**	135°<br>218°	343°<br>54°	*****<br>*****	311°<br>3°	107°<br>180°	193°<br>98°	114°<br>197°	304°<br>9°	196°<br>215°	295°<br>22°	144°<br>249°	118°<br>301°
**RT (z)**	5.483	7.103	0.366	12.43	7.119	0.61	5.834	9.154	0.303	5.157	3.703	1.259
**RT (p)**	0.003	5.2E-4	0.701	1.1E-6	5.5E-4	0.548	0.003	6.9E-5	0.741	0.005	0.023	0.287
**MMRT (R*)**	1.471	1.563	0.581	2.044	1.526	0.404	1.57	1.592	0.454	1.493	1.174	0.407
**MMRT (p)**	< 0.005	< 0.001	0.1<p<0.5	< 0.001	< 0.001	0.5<p<0.9	< 0.001	< 0.001	0.5<p<0.9	< 0.005	< 0.025	0.5<p<0.9

A. Spring 2018 natural orientation
B. Spring 2018 180° rotation
C. Spring 2018 randomised
D. Autumn 2018 natural orientation
E. Autumn 2018 180° rotation
F. Autumn 2018 randomised
G. Spring 2019 natural orientation
H. Spring 2019 180° rotation
I. Spring 2019 randomised
J. Autumn 2019 natural orientation
K. Autumn 2019 180° rotation
L. Autumn 2019 randomised

n: number of moths tested
MV: Mean vector, **μ** = vector direction, **r** = vector length (0<r<1)
GMV: Grand mean vector, **α** = vector direction
CV: Circular variance
CSD: Circular standard deviation
95% CI: 95% Confidence interval (both angular limits (+/-) shown)
99% CI: 99% Confidence interval (both angular limits (+/-) shown)
RT: Rayleigh Test
z: Rayleigh z statistic = nr^2
p: Probability of obtaining observed results, assuming null hypothesis is true
R*: Directedness of the MV for a population of tested moths
MMRT: Moore's Modified Rayleigh Test
