# Supplementary material for: Bogong moths use a stellar compass for long-distance navigation at night
Source: Nature. 2025 Jun 18;643(8073):994–1000. doi: 10.1038/s41586-025-09135-3 (PMC12286839; doi:10.1038/s41586-025-09135-3)
Supplement: Supplementary file 2 — Reporting Summary [file 41586_2025_9135_MOESM2_ESM.pdf]

nature portfolio

Corresponding author(s): Eric Warrant

Last updated by author(s): April 14, 2025

Reporting Summary

Nature Portfolio wishes to improve the reproducibility of the work that we publish. This form provides structure for consistency and transparency in reporting. For further information on Nature Portfolio policies, see our Editorial Policies and the Editorial Policy Checklist.

Statistics

For all statistical analyses, confirm that the following items are present in the figure legend, table legend, main text, or Methods section.

n/a	Confirmed	
☐	☒	The exact sample size (*n*) for each experimental group/condition, given as a discrete number and unit of measurement
☐	☒	A statement on whether measurements were taken from distinct samples or whether the same sample was measured repeatedly
☐	☒	The statistical test(s) used AND whether they are one- or two-sided<br>*Only common tests should be described solely by name; describe more complex techniques in the Methods section.*
☒	☐	A description of all covariates tested
☐	☒	A description of any assumptions or corrections, such as tests of normality and adjustment for multiple comparisons
☐	☒	A full description of the statistical parameters including central tendency (e.g. means) or other basic estimates (e.g. regression coefficient) AND variation (e.g. standard deviation) or associated estimates of uncertainty (e.g. confidence intervals)
☐	☒	For null hypothesis testing, the test statistic (e.g. *F*, *t*, *r*) with confidence intervals, effect sizes, degrees of freedom and *P* value noted<br>*Give P values as exact values whenever suitable.*
☒	☐	For Bayesian analysis, information on the choice of priors and Markov chain Monte Carlo settings
☒	☐	For hierarchical and complex designs, identification of the appropriate level for tests and full reporting of outcomes
☒	☐	Estimates of effect sizes (e.g. Cohen's *d*, Pearson's *r*), indicating how they were calculated

Our web collection on statistics for biologists contains articles on many of the points above.

Software and code

Policy information about availability of computer code

Data collection	Custom-written MATLAB code (versions 2019a and 2022b) to control magnetic & stellar simulation, USB1 Explorer encoder software (version 1.07, US Digital) to track moths, Spike2 (version 8.03, Cambridge Electronic Design) to collect electrophysiology data.
Data analysis	Custom-written MATLAB code (versions 2019a and 2022b) to analyse moth trajectories and electrophysiology data, MS Excel (MSO 2019), R (version 3.6.1) and Oriana (version 4, 2011) to perform the statistics. Code available: github.com/stanleyheinze/Starry_sky_code

For manuscripts utilizing custom algorithms or software that are central to the research but not yet described in published literature, software must be made available to editors and reviewers. We strongly encourage code deposition in a community repository (e.g. GitHub). See the Nature Portfolio guidelines for submitting code & software for further information.

Data

Policy information about availability of data

All manuscripts must include a data availability statement. This statement should provide the following information, where applicable:

- Accession codes, unique identifiers, or web links for publicly available datasets
- A description of any restrictions on data availability
- For clinical datasets or third party data, please ensure that the statement adheres to our policy

The experimental data that support the findings of Figures 2, 3, 4 and 5 of this study are available in Figshare with the identifier https://doi.org/10.6084/m9.figshare.25780197.v2. The experimental data that support the findings of Figures 4 and 5 can also be found in the Extended Data.

Research involving human participants, their data, or biological material

Policy information about studies with human participants or human data. See also policy information about sex, gender (identity/presentation), and sexual orientation and race, ethnicity and racism.

Reporting on sex and gender	Not applicable
Reporting on race, ethnicity, or other socially relevant groupings	Not applicable
Population characteristics	Not applicable
Recruitment	Not applicable
Ethics oversight	Not applicable

Note that full information on the approval of the study protocol must also be provided in the manuscript.

Field-specific reporting

Please select the one below that is the best fit for your research. If you are not sure, read the appropriate sections before making your selection.

☒ Life sciences ☐ Behavioural & social sciences ☐ Ecological, evolutionary & environmental sciences

For a reference copy of the document with all sections, see nature.com/documents/nr-reporting-summary-flat.pdf

Life sciences study design

All studies must disclose on these points even when the disclosure is negative.

Sample size	As we were dealing with wild-caught migratory insects that were caught over periods of several weeks and were difficult to catch in large numbers, we simply used as many individuals as we could. In our behavioural work, we routinely obtained significant results even after a relatively low number of individuals were tested (around 15-20). However, since we wished to make sure we could replicate our results over at least four migratory seasons, our sample sizes were typically around 50-70 individuals.
Data exclusions	From our Methods section: "Moths chosen for analysis were required to fulfil three ante hoc criteria, two prior to the experiment and one during the experiment: (1) the tethering stalk was perfectly vertical, (2) wing flapping was vigorous and its amplitude was large and equal for both wings (indicating that the contact cement had not interfered with the wings), and (3) that the moth flew continuously for the full 5 min. For the last criterion, if a moth stopped flying, the arena was gently tapped in order to stimulate the moth to continue flight behaviour. A moth that stopped flying 4 times was rejected and the recording aborted."
Replication	We replicated our behavioural results over 2 spring migratory seasons and 2 autumn migratory seasons. The replication was so good that we pooled the data from the two spring migratory seasons into a single data set. The same was true for the two autumn migratory seasons. With regards to the electrophysiological data, since each recording was a unique observation, replication was not possible. This is due to the stochastic nature of intracellular recordings - single neurons were penetrated randomly from target brain regions that contain many thousands of cells.
Randomization	Allocation of moths to experiments was random. Each afternoon approximately 20-30 moths were collected randomly from our store of moths and a tether was glued to the back of each of them for behavioural experiments. These moths were then used for experiments on the same night. Afterwards their stalks were removed and they were released into the wild. In parallel, on the same afternoons, 3-5 moths were similarly collected (i.e. randomly) and prepared for electrophysiological experiments. Moths used for these experiments were sacrificed at the conclusion of the experiment by swift removal of the head using a razor blade.
Blinding	Our study was not blinded as a single individual carried out the experiment and it was impossible to hide the experimental condition applied.

Reporting for specific materials, systems and methods

We require information from authors about some types of materials, experimental systems and methods used in many studies. Here, indicate whether each material, system or method listed is relevant to your study. If you are not sure if a list item applies to your research, read the appropriate section before selecting a response.

Materials & experimental systems

n/a	Involved in the study
☒	☐ Antibodies
☒	☐ Eukaryotic cell lines
☒	☐ Palaeontology and archaeology
☐	☒ Animals and other organisms
☒	☐ Clinical data
☒	☐ Dual use research of concern
☒	☐ Plants

Methods

n/a	Involved in the study
☒	☐ ChIP-seq
☒	☐ Flow cytometry
☒	☐ MRI-based neuroimaging

Animals and other research organisms

Policy information about studies involving animals; ARRIVE guidelines recommended for reporting animal research, and Sex and Gender in Research

Laboratory animals	The study did not involve laboratory animals.
Wild animals	The Australian Bogong moth Agrotis infusa, wild caught during their migration to the Australian Alps in spring (age: ca. 5-6 months old) and from the Australian Alps in autumn (age: ca. 8-9 months old). Migrating moths were caught using light traps between 9 pm and 11 pm in alpine areas. They were placed in individual plastic containers (one moth per container) and transported back to the lab (70 km away) in a car. Following the single night of behavioural experiments that each moth was subjected to, moths were released back to the wild (following removal of the tethering stalk). This release was made near the lab at a location that is close to the natural migratory route. Moths used for electrophysiological experiments were sacrificed at the conclusion of the experiment by swift removal of the head using a razor blade.
Reporting on sex	We always noted the sex of each moth we tested, however as there was no statistical difference in the results obtained from males and females the data was pooled.
Field-collected samples	Our study involved lab experiments on wild-caught moths (see above). Prior to experiments moths were housed in small cylindrical plastic containers with screw-on lids (ca. 5 cm diameter and 7 cm high) and kept in cool dim conditions with a natural but maximally dim light cycle. Moths were not held like this for more than 3 days and were fed daily with an earbud soaked in honey solution.
Ethics oversight	No ethical approval is required for working on insects. However, the work was performed under a Scientific License issued by the Australian Government.

Note that full information on the approval of the study protocol must also be provided in the manuscript.

Plants

Seed stocks	Not applicable
Novel plant genotypes	Not applicable
Authentication	Not applicable
